# Non-SMC condensin I complex subunit D2 (NCAPD2) reveals its prognostic and immunologic features in human cancers

**DOI:** 10.18632/aging.204904

**Published:** 2023-07-26

**Authors:** Xiaoying Dong, Ting Liu, Zhizhao Li, Yongzhen Zhai

**Affiliations:** 1 Department of Infectious Disease, Shengjing Hospital of China Medical University, Shenyang 110004, People’s Republic of China; 2 Department of Pathology, Beijing Ditan Hospital, Capital Medical University, Chaoyang 100015, Beijing, People’s Republic of China; 3 Department of Cardiovascular, Beijing Ditan Hospital, Capital Medical University, Chaoyang 100015, Beijing, People’s Republic of China

**Keywords:** NCAPD2, pan-cancer, prognosis, immune analysis

## Abstract

Non-SMC condensin I complex subunit D2 (NCAPD2) is overexpressed in some malignant tumors. However, there are few studies on the function of NCAPD2 in pan-cancer. We used the Cancer Genome Atlas (TCGA), Genotype-Tissue Expression (GTEx), Human Protein Atlas (HPA), and UALCAN to analyze NCAPD2 expression and promoter methylation levels in 33 tumors and normal samples. We performed immunohistochemistry (IHC) on liver cancer and corresponding normal tissues to examine NCAPD2 protein expression in LIHC. Kaplan-Meier survival and univariate regression analyses were performed to explore the pan-cancer clinical significance of NCAPD2. Moreover, correlative analysis between NCAPD2 expression and clinical characteristics, immune cell infiltration, immune checkpoints, immune regulators, tumor mutation burden (TMB), microsatellite instability (MSI), ribonucleic acid (RNA) methylation regulators, and drug sensitivity was conducted using data from TCGA. We also investigated the effects of NCAPD2 expression on immunotherapy efficacy and prognosis. Gene set enrichment analysis (GSEA) was conducted using NCAPD2. Bioinformatic analysis showed that NCAPD2 was overexpressed in most tumors and correlated with the clinical characteristics of some cancers. IHC results demonstrated that NCAPD2 protein expression was higher in LIHC than in normal liver. NCAPD2 expression was linked with T stage, clinical stage, and histologic grade in LIHC. Overexpression of NCAPD2 resulted in poor overall survival, and disease-specific survival in adrenocortical carcinoma, kidney renal papillary cell carcinoma, brain lower grade glioma, liver hepatocellular carcinoma, lung adenocarcinoma, mesothelioma, pancreatic adenocarcinoma, sarcoma, skin cutaneous melanoma, and uterine corpus endometrial carcinoma. NCAPD2 was considered an independent biomarker by Cox regression in LIHC. The time ROC curve demonstrated that the survival rate of 1-, 3-, and 5-year OS and DSS in LIHC was above 0.6. The expression of NCAPD2 was significantly correlated with immune cell infiltration, immune checkpoints, TMB, MSI, and RNA methylation regulators in several tumors. NCAPD2 had a high predictive value for immunotherapy efficiency in certain tumors. In our study, drugs sensitive to NCAPD2 protein were screened by sensitivity analysis. GSEA analysis showed that NCAPD2 mainly participated in the G2M checkpoint, mitotic spindle, and KRAS-signaling. NCAPD2 may act as a prognostic molecular marker in most cancers.

## INTRODUCTION

Cancer poses a significant threat to human health. According to the World Health Organization’s International Agency for Research on Cancer, there will be 19.3 million new cancer cases and 10 million cancer deaths in 2020 worldwide, seriously endangering human health. In addition, cancer patients usually have poor prognosis and low survival rates, which directly affect their quality of life [1]. Tumor formation is a complex process involving cancer cell proliferation, anti-apoptosis, enhanced angiogenesis, immune escape, and other processes. Recently, pan-cancer studies have become prominent, contributing to the exploration of the molecular mechanisms of different tumors. This study focused on non-SMC condensin I complex subunit D2 (NCAPD2) and investigated its effect on the pathogenesis and development of different tumors using pan-cancer analysis.

The condensin complex plays a vital role in chromosome condensation in eukaryotes [2]. In vertebrates, there are two types of condensin complexes: condensin complex I and condensin complex II. NCAPD2 is a subunit of condensin complex I and is mainly involved in the condensation and separation of chromatin during mitosis [3, 4]. Several studies have suggested that NCAPD2 deletion affects the expression of transcription genes during mitotic interphase. In addition, the knockout of NCAPD2 inhibits the proliferation and invasion of triple-negative breast cancer (TNBC) cells [5]. The NCAPD2 gene is positively correlated with the risk of Parkinson’s disease in the Han population and acts as a latent genetic marker for sporadic Parkinson’s [6]. Previous studies on NCAPD2 are limited to certain types of tumors, and pan-cancer NCAPD2 expression and correlation analysis have rarely been discussed. In this study, we employed multiple databases and pan-cancer analyses to study the function of NCAPD2 in the prognosis and immunity of multiple tumors. We also investigated the correlation between NCAPD2 expression and clinical features, tumor mutation burden (TMB), microsatellite instability (MSI), tumor microenvironment (TME), immune checkpoints, deoxyribonucleic acid (DNA), ribonucleic acid (RNA) methylation, and drug sensitivity to discuss the molecular mechanisms of NCAPD2 in carcinogenesis, clinical outcomes, and immunotherapy for various human tumors.

## MATERIALS AND METHODS

### Gene expressions analysis and the correlation analysis with clinical features

The differential expression of NCAPD2 in tumors and normal tissues were studied using the expression profile data of 33 tumors obtained from the Cancer Genome Atlas (TCGA) and the Genotype-Tissue Expression (GTEx) database. Clinical information was acquired from TCGA. The abbreviations of 33 tumors are shown in Supplementary Table 1. Subsequently, the Clinical Proteomics Tumor Analysis Consortium (CPTAC) dataset from the UALCAN database was applied to analyze NCAPD2 protein expression in breast cancer, colon cancer, uterine body endometrial cancer, lung adenocarcinoma, and ovarian cancer. The Human Protein Atlas (HPA) is an online database that provides tissue and cell distribution information for all 24,000 human proteins. Immunohistochemical images of the NCAPD2 protein in four types of tumors and corresponding normal tissues were acquired from the HPA database. Moreover, we applied the ROC curve to evaluate the diagnostic value of NCAPD2 in cancers and normal tissues using the pROC package in R. Wilcoxon test was used to study the correlation between NCAPD2 and clinical characteristics, including race, T stage, N stage, and pathological stage. Race was divided into Asian and non-Asian groups. Non-Asian people included black or African American, and White. The T stage was separated into the T1 + T2 and T3 + T4 groups, and the N stage was divided into N0, and N1&N2&N3 groups. The pathological stage was classified into stage I+II and stage III+IV stage groups.

### Prognosis analysis

The patients were divided into low- and high-expression groups based on the median value of NCAPD2, and survival differences of different cancer patients were discussed using the R language’s “survminer” package and “survival” package. Kaplan-Meier survival was used to explore the impact of NCAPD2 expression on overall survival (OS). For survival analysis, the effects of NCAPD2 on OS, and disease-specific survival (DSS) of 33 patients with cancer were studied by univariate regression analysis and visualized using forest plots.

### Immunity analysis

We used the Tumor Immune Estimation Resource (TIMER) database to study the relationship between NCAPD2 expression in various tumors and six types of immune cell infiltration, including B cells, CD8+T cells, CD4+T cells, macrophages, neutrophils, and dendritic cells [7]. ESTIMATE is an algorithmic tool that estimates the proportion of stromal cells and immune cell infiltration as well as the content of tumor cells in malignant tumor tissues according to gene expression data [8]. The association between NCAPD2 and immune regulatory factors, including immunosuppressants, immune stimulators, major histocompatibility complex (MHC) molecules, and chemokines, was assessed using Spearman’s correlation analysis. Immune checkpoint is closely related to tumor proliferation, invasion, metastasis, and prognosis, which is a good target for tumor treatment. Finally, we explored the correlation between NCAPD2 expression and eight common immune checkpoints (ICs) by Spearman correlation analysis.

### TMB and MSI analysis

TMB and MSI are two highly effective biomarkers for tumor immunotherapy that have attracted clinical attention. TMB is defined as the incidence of somatic mutations per million bases. Previous studies have confirmed that patients with high TMB are responsive to immunotherapy and are emerging biomarkers that are sensitive to immune checkpoint inhibitors [9–15]. MSI refers to the emergence of new microsatellite alleles due to the insertion or deletion of repetitive units at microsatellite loci in tumors. MSI results from functional defects in DNA mismatch repair of tumors. Similar to TMB, it has been demonstrated to be an important biomarker for the clinical potency of anti-PD-1/PD-L1 immune checkpoint inhibitors [16]. Therefore, correlation analysis with TMB and MSI is beneficial for the evaluation of immunotherapy response.

### DNA and RNA methylation analysis

DNA methylation and RNA methylation are two types of epigenetic modifications that play a major role in the progression of malignant tumors and may be significant markers for tumor diagnosis, treatment, and prognosis [17–19]. We analyzed promoter methylation levels in cancer and normal tissues using the UALCAN database [20]. RNA methylation mainly involves m6A, m5C, and m1A, which are composed of writers, readers, and erasers. Spearman’s correlation was used to investigate the relationship between NCAPD2 expression and m6A, m5C, and m1A regulatory genes.

### Gene set enrichment analysis

To study the biological function and oncogenic pathways involved in NCAPD2, we downloaded the “gmt” file of the hallmark gene sets (h.all.v7.4. symbols. gmt) from MSigDB (https://www.gsea-msigdb.org/gsea/msigdb/index.jsp), which contains 50 marker gene sets. We then divided NCAPD2 into low- and high-expression groups, and performed gene set enrichment analysis (GSEA) in 33 tumors by the R package “cluster Profiler” [21]. The results were visualized using heat maps. Statistical significance was set at *P* < 0.05 and FDR < 0.25.

### Response to immunotherapy analysis

TISIDB (http://cis.hku.hk/TISIDB/) is a web portal that provides the differential expression of NCAPD2 in responders and nonresponders to immunotherapy. Tumor Immune Dysfunction and Exclusion (TIDE) (http://tide.dfci.harvard.edu) is a web platform for inferring the gene function of tumor immunity and evaluating biomarkers to predict the clinical response to immune checkpoint blocks (ICB). We used TIDE to compare the predictive power of NCAPD2 with that of other published biomarkers for response outcomes and overall survival. Area under the curve (AUC) values were used to test the predictive power of ICB responses in the different treatment groups.

### Drug sensitivity analysis

We used GSCALite (http://bioinfo.life.hust.edu.cn/web/GSCALite/) to analyze gene expression and drug sensitivity [22]. GSCALite can provide 750 small-molecule drugs from GDSC and CTRP and uses gene expression data to mine valuable small-molecule drugs associated with it.

### Immunohistochemistry (IHC)

We collected twenty cases of liver cancers and ten normal tissues from Shengjing Hospital of China Medical University for IHC. Among them, there were 15 males and 5 females, aged from 30 years to 70 years, twelve patients with stage I+II and eight patients with stage III+IV, and survival times ranging from 35 to 1580 days. The study was authorized by local institutional review boards, and written informed consent was obtained from all patients before surgery. The slides were deparaffinized, antigen repaired with antigen repair solution and blocked with animal serum. Subsequently, NCAPD2 rabbit polyclonal antibody (1:100, Proteintech Company, USA) was added to the slides and left overnight at 4° C. The next day, sheep anti-rabbit IgG polymer (PV-6000, Zhongshan Jinqiao Biotechnology Company, Beijing, China) was added for half an hour at room temperature, followed by DAB for 3 min. Then, they were counterstained with hematoxylin and dehydrated, and sealed with neutral glue. NCAPD2 protein expression in different cancers and normal samples was detected using IHC. NCAPD2 protein expression was semi-quantitatively calculated by multiplying expression intensity by expression area. We randomly selected 10 different fields of view and observed them under a 200X microscope. The expression intensity ranged from 0 to 3 which were negative, weak, moderate, and strong staining. Expression area scores ranged from 0 to 4, representing < 5%, 6-25%, 26-50%, 51-75%, and > 75%, respectively. The positive degree of staining was defined as weak positive (1-3, +); moderate positive (4-6, ++); strong positive (7-12, +++).

### Statistical analysis

The differences in gene expression among various cancer types and normal samples in the TCGA and GTEx databases were studied using the Wilcoxon test. Wilcoxon test was also used to explore the association between NCAPD2 and patients’ clinical features. Kaplan-Meier survival and univariate Cox regression were used to study the impact of NCAPD2 on the prognosis of tumors using R language’s survminer and survival packages. The receiver operating characteristic (ROC) curve was applied using the pROC package. The relationship of NCAPD2 with TME, immune regulators, TMB and MSI, RNA methylation-regulated genes, and drug sensitivity was explored by Spearman correlation analysis. Statistical significance was set at P < 0.05.

### Data availability

The data included in the current study were obtained from TCGA database, GTEx, HPA, TIMER, Cbioportal, UALCAN, TISIDB, TIDE, and GSCALite. This article contains data that supports the findings of this study.

## RESULTS

### NCAPD2 expression is overexpressed in most cancers and related to clinical pathology

**Figure 1:**
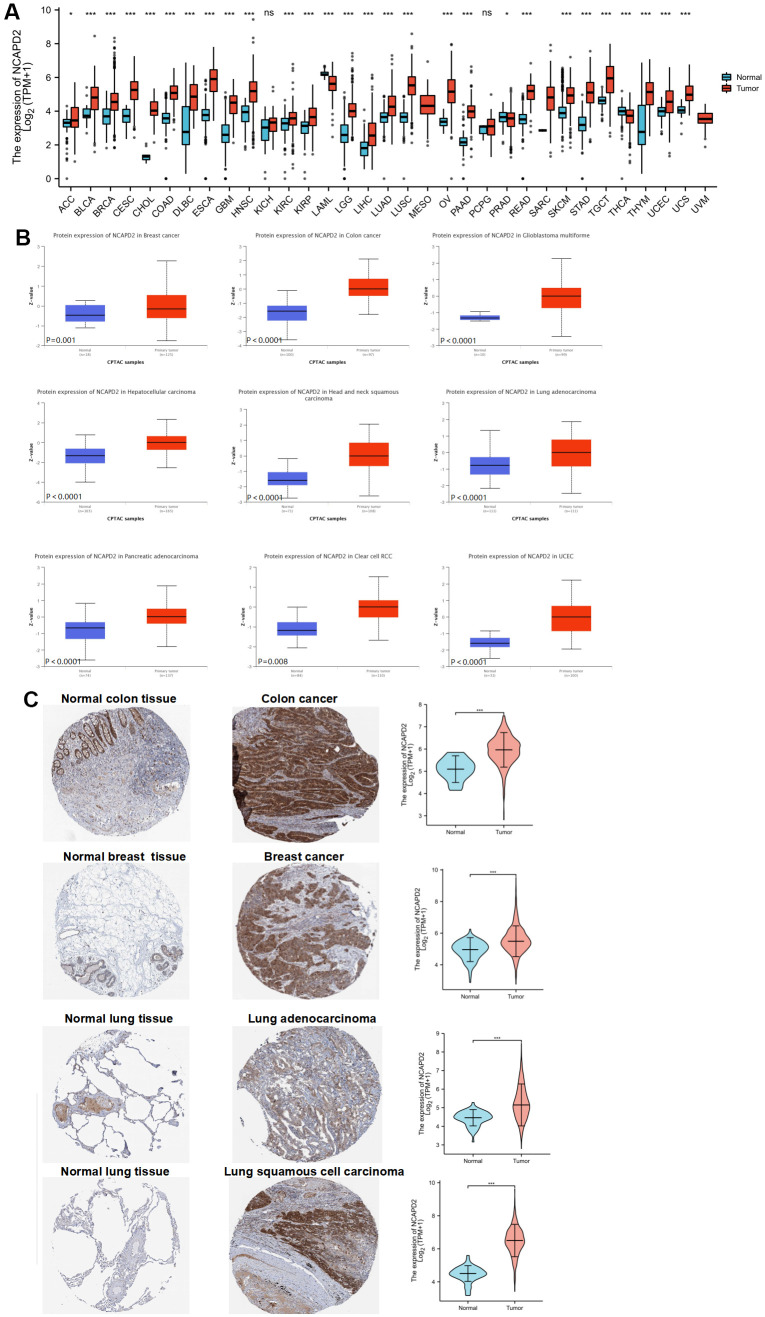
NCAPD2 expression in different databases (***P < 0.001,**P < 0.01,*P < 0.05, ns no significance). (**A**) NCAPD2 expression from TCGA and GTEx. (**B**) NCAPD2 expression in CPTAC for some cancers. (**C**) NCAPD2 expression was higher in colon cancer, breast cancer, lung adenocarcinoma and lung squamous cell carcinoma than in corresponding normal tissues.

We downloaded the NCAPD2 expression profile data of 33 cancers and corresponding normal tissue samples from TCGA and GTEx. The results of the Wilcoxon test analysis suggested that NCAPD2 was highly expressed in 25 tumors, but low in LAML, PRAD, and THCA relative to normal tissues (Figure 1A). In addition, NCAPD2 protein expression in BRCA, COAD, GBM, LIHC, HNSCC, LUAD, PAAD, KIRC, and UCEC from the CPTAC database was higher than that in the normal tissues (Figure 1B). According to the HPA database, NCAPD2 protein expression in colon cancer, breast cancer, lung adenocarcinoma, and lung squamous cell carcinoma was higher than that in the corresponding normal tissues (Figure 1C). Moreover, we generated ROC curves to evaluate the diagnostic efficacy of NCAPD2 in pan-cancer and normal samples. The AUC of NCAPD2 in 20 types of cancer was > 0.8, indicating that NCAPD2 had high diagnostic accuracy in distinguishing cancer from normal tissues (Supplementary Figure 1).

**Figure 2:**
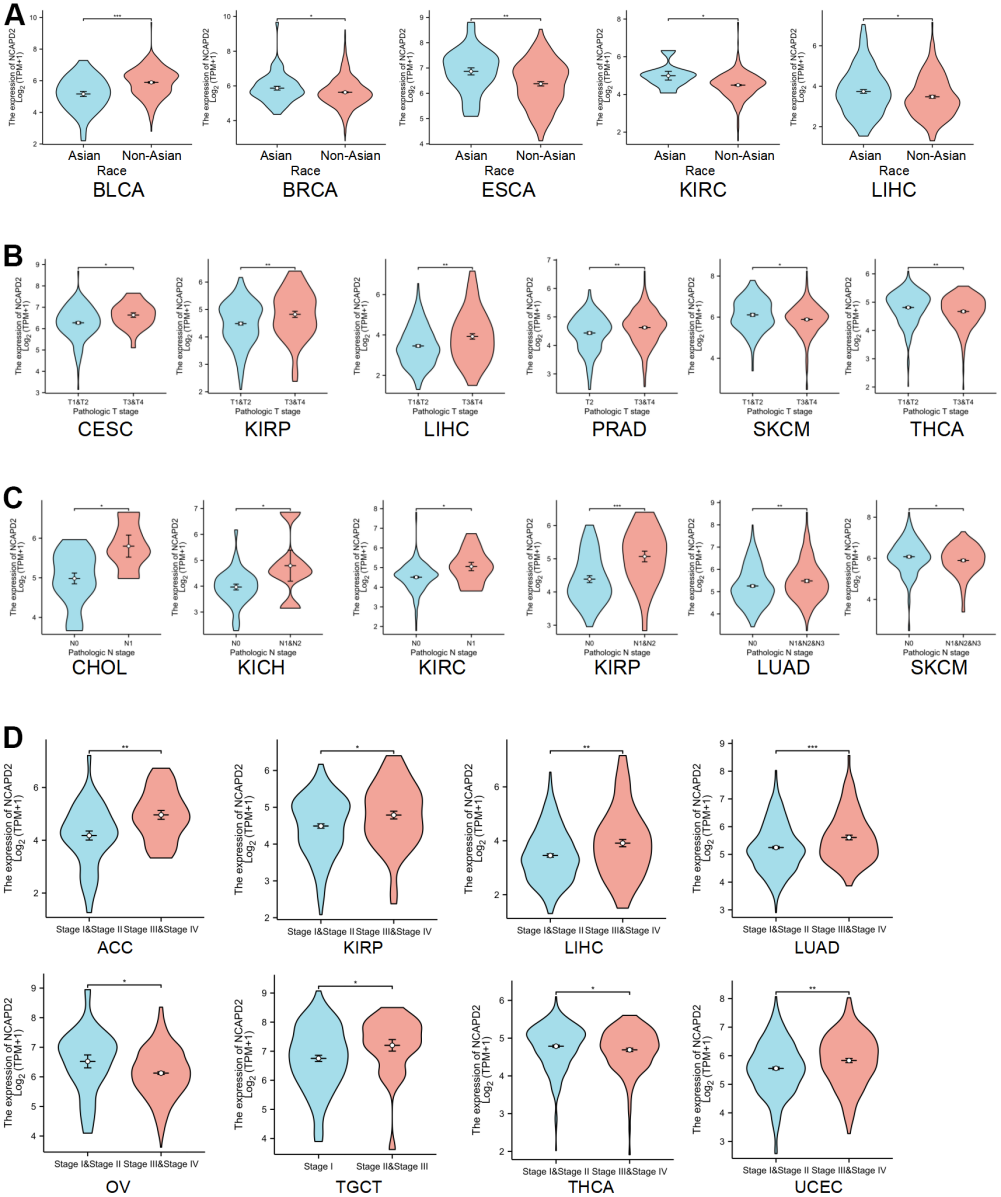
**Correlation between NCAPD2 expression and clinical features.** (**A**) The correlation between NCAPD2 expression and race. (**B**) The correlation between NCAPD2 expression and T stage. (**C**) The correlation between NCAPD2 expression and N stage. (**D**) The correlation between NCAPD2 expression and clinical stage. (***P < 0.001,**P < 0.01,*P < 0.05).

We analyzed the association between NCAPD2 and clinical features, including race, T stage, and N stage. For BLCA, NCAPD2 expression was higher in the non-Asian group than in the Asian group. Conversely, NCAPD2 expression was lower in the non-Asian groups than in the Asian groups for BRCA, ESCA, KIRC, and LIHC (Figure 2A). NCAPD2 had higher expression at higher T stages for CESC, KIRP, LIHC, and PRAD and lower expression at higher T stages for SKCM and THCA (Figure 2B). The expression of NCAPD2 in cancer with lymph node metastasis was significantly higher than that without lymph node metastasis in CHOL, KICH, KIRC, KIRP, and LUAD and significantly lower in SKCM (Figure 2C). As shown in Figure 2D, NCAPD2 was correlated with the pathological stage in eight tumors. NCAPD2 expression increased with the clinical stage in ACC, KIRP, LIHC, LUAD, TGCT, and UCEC. NCAPD2 expression was higher in stage III+IV in ACC, KIRP, LIHC, LUAD, TGCT, and UCEC than in stage I+II. In OV and THCA, NCAPD2 expression decreased with tumor progression.

### NCAPD2 overexpression is associated with adverse outcomes in cancers

**Figure 3:**
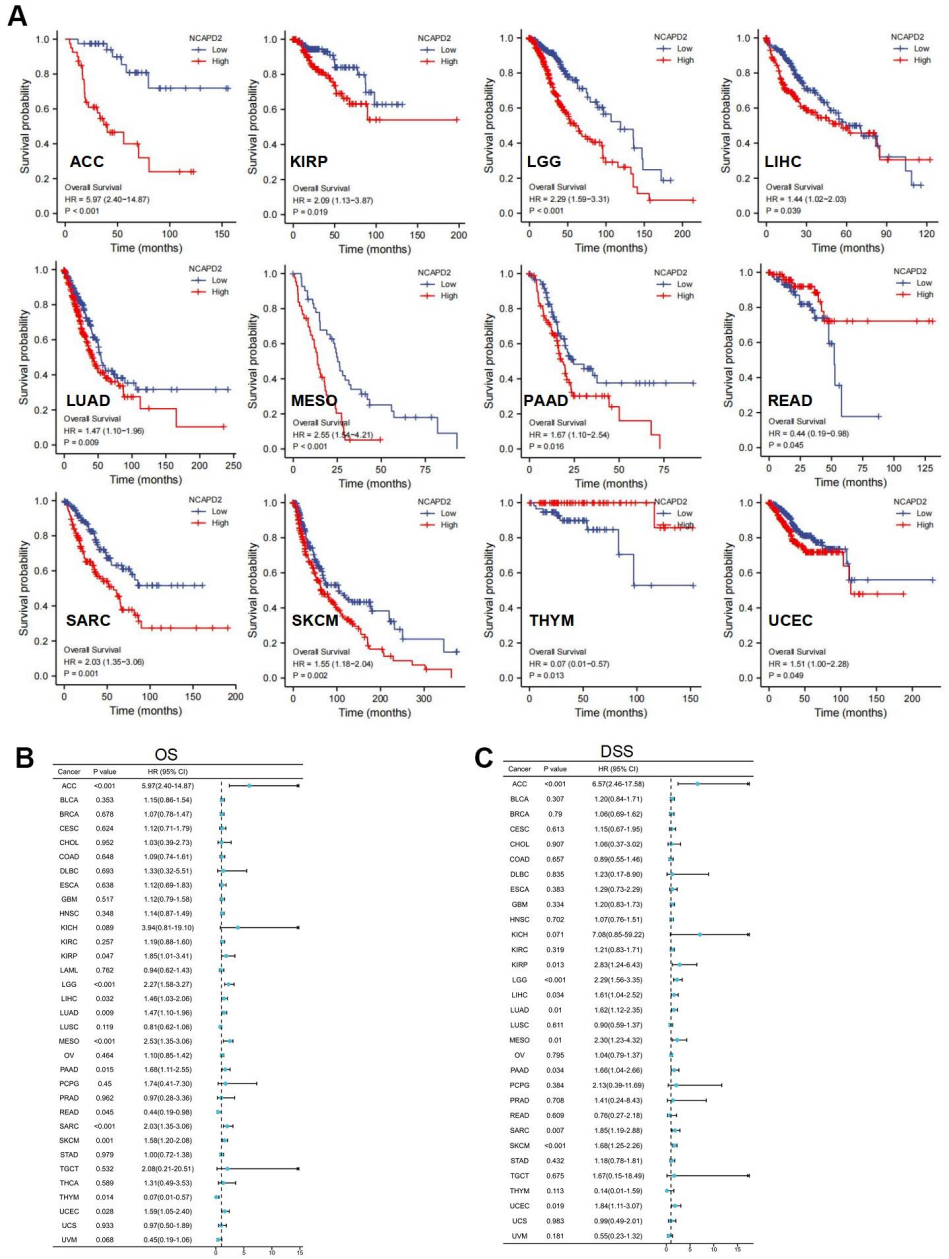
**NCAPD2 prognosis in 33 cancers was analyzed using Kaplan-Meier survival and univariate Cox analysis.** (**A**) Kaplan-Meier survival curve of NCAPD2 in high- and low-expression groups for OS in ACC, KIRP, LGG, LIHC, LUAD, MESO, PAAD, READ, SARC, SKCM, THYM, UCEC, and UVM. Univariate Cox regression analysis comparing NCAPD2 expression and (**B**) overall survival, and (**C**) disease-specific survival in 33 cancers.

Kaplan-Meier survival curves revealed that NCAPD2 in the high-expression group had worse OS for ACC, KIRP, LGG, LIHC, LUAD, MESO, PAAD, SARC, SKCM, and UCEC but better OS for READ, and THYM (Figure 3A). We selected OS, and DSS to investigate the prognostic value of NCAPD2 in different cancers. OS analysis showed that NCAPD2 was significantly associated with ACC, KIRP, LGG, LIHC, LUAD, MESO, PAAD, READ, SARC, SKCM, THYM, and UCEC. Overexpression of NCAPD2 was a hazard factor for ACC, KIRP, LGG, LIHC, LUAD, MESO, PAAD, SARC, SKCM, and UCEC and a protective factor for READ and THYM (Figure 3B). DSS results showed that NCAPD2 was a risk factor for ACC, KIRP, LGG, LIHC, LUAD, MESO, PAAD, SARC, SKCM, and UCEC (Figure 3C). Therefore, OS and DSS results showed that NCAPD2 in ACC, KIRP, LGG, LIHC, LUAD, MESO, PAAD, SARC, SKCM, and UCEC had poor outcomes.

### Correlation analysis of NCAPD2 expression with tumor microenvironment (TME) and immune checkpoints in pan-cancer

**Figure 4:**
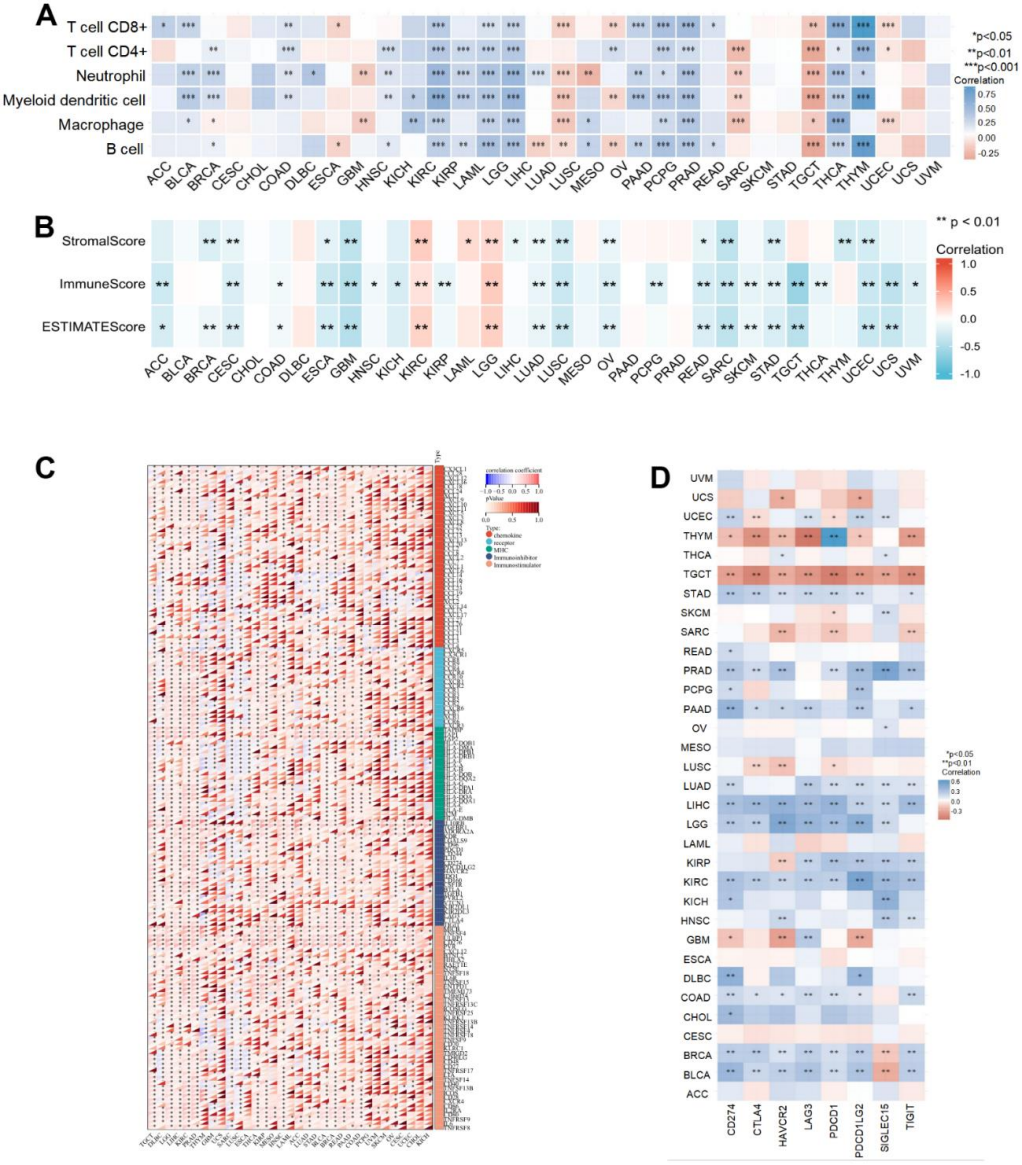
**The relationship of NCAPD2 with immunity in pan-cancer.** (**A**) The correlation between NCAPD2 and immune cell infiltration by TIMER database. (**B**) The correlation between NCAPD2 and stromal score, immune score, and estimate score by ESTIMATE algorithm. (**C**) The correlation between NCAPD2 and immune regulatory genes. (**D**) The correlation between NCAPD2 and immune checkpoints. (***P < 0.001, **P < 0.01,*P < 0.05).

Tumor immune cell infiltration is also involved in cancer progression. We used the TIMER database to investigate the association between NCAPD2 expression and six immune cells from 33 cancers. NCAPD2 expression was positively correlated with the infiltration of six immunocytes in KIRC, LAML, LGG, PCPG, PRAD, and THCA, but a negative association between NCAPD2 expression and immunocyte infiltration was noted in TGCT. In CESC, CHOL, SKCM, STAD, UCS, and UVM, NCAPD2 was not related to immunocyte infiltration. NCAPD2 was associated with one or more immune cell infiltrations in other cancers (Figure 4A). In addition, the relationship between NCAPD2 and the immune score, stromal score, and estimate score for 33 tumors was discussed using the ESTIMATE algorithm. There was a negative correlation between NCAPD2 and stromal, immune, and estimated scores in CESC, ESCA, GBM, LUAD, LUSC, OV, READ, SARC, STAD, and UCEC. Conversely, a positive correlation was noted in KIRC and LGG. Except for BLCA, CHOL, DLBC, MESO, PAAD, and PRAD, NCAPD2 was associated with the stromal score, immune score, or estimate score in 15 other tumors. This suggested that NCAPD2 plays a role in the TME (Figure 4B).

Furthermore, we evaluated the relationship between NCAPD2 and immunomodulators, including 41 chemokines, 18 receptors, 12 MHC, 24 immunoinhibitors, and 46 immunostimulators. NCAPD2 expression positively correlated with multiple immune modulators in most tumors (Figure 4C). The emergence of immune checkpoints (ICs) has led to breakthrough progress in immunotherapy, and its inhibitors have been used in the treatment of some tumors. Our results revealed that NCAPD2 was positively correlated with eight ICs in LIHC and KIRC but negatively correlated with TGCT. In BRCA and BLCA, there was a positive relationship between NCAPD2 and CD274, CTLA4, HAVCR2, LAG3, PDCD1, PDCD1LG2, and TIGIT and a negative relationship with SIGLEC15. NCAPD2 was not associated with the eight ICs for ACC, CESC, ESCA, LAML, MESO, and UVM. NCAPD2 expression was positively or negatively correlated with one or more ICs in the other 22 tumors (Figure 4D).

### NCAPD2 expression is correlated with TMB and MSI

**Figure 5:**
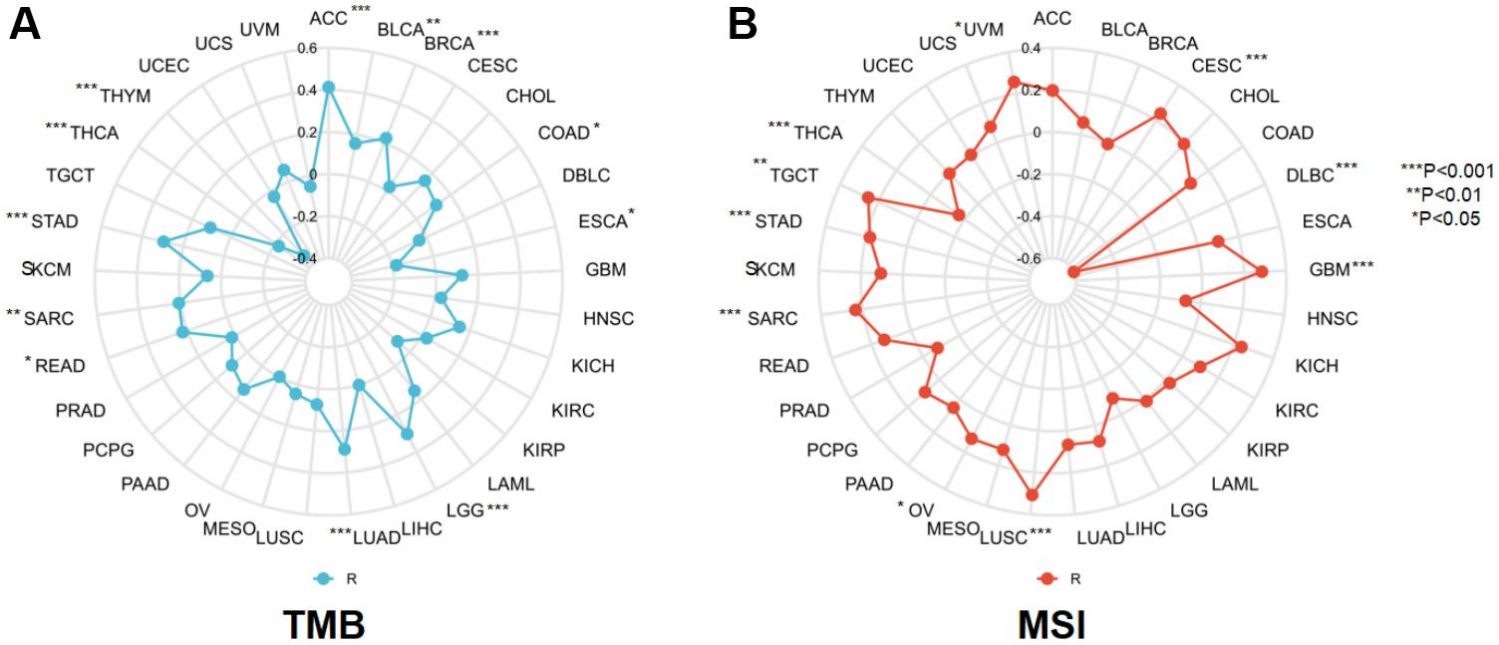
The correlation between NCAPD2 and (**A**) TMB and (**B**) MSI in pan-cancer. (***P < 0.001,**P < 0.01,*P < 0.05).

TMB and MSI are two novel markers of immunotherapeutic response, making it necessary to investigate their association with NCAPD2 in various tumors. NCAPD2 had a significantly positive correlation with TMB in ACC, BLCA, BRCA, COAD, LGG, LUAD, READ, SARC, and STAD, with the largest correlation coefficient in ACC. In ESCA, THCA, and THYM, NCAPD2 was negatively correlated with TMB, with the strongest negative correlation observed in THYM (Figure 5A). For MSI, NCAPD2 was significantly positively associated with CESC, GBM, LUSC, OV, SARC, STAD, TGCT, and UVM and negatively associated with DLBC and THCA (Figure 5B). These results indicated that the correlative study of NCAPD2 with TMB and MSI provided a basis for immunotherapy.

### NCAPD2 expression is linked with DNA and RNA methylation

**Figure 6:**
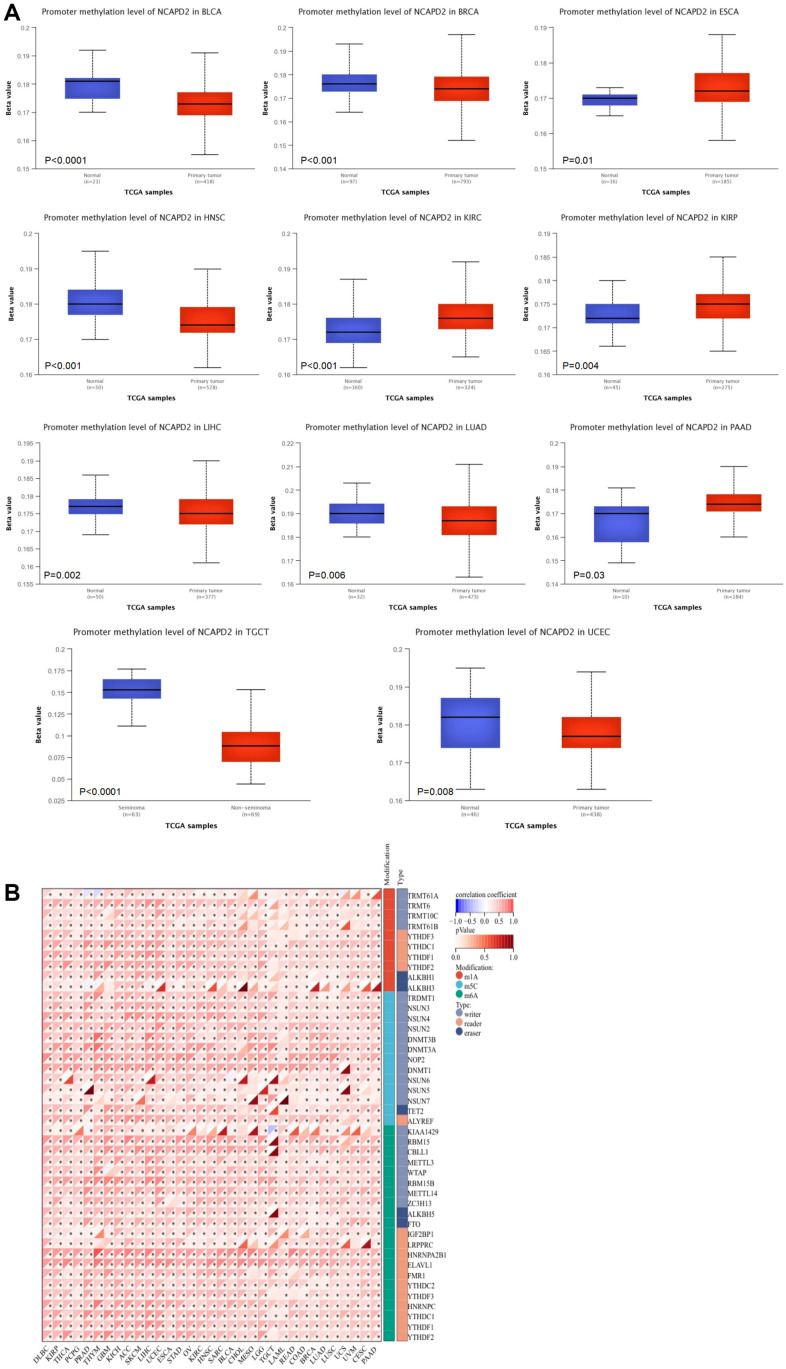
**The correlation between NCAPD2 expression and methylation.** (**A**) The promoter methylation level of NCAPD2 in some cancers. (**B**) The correlation between NCAPD2 and RNA methylation regulators. (***P < 0.001,**P < 0.01,*P < 0.05).

Using the UALCAN database, we found that, compared with normal samples, the level of NCAPD2 promoter methylation was lower in BLCA, BRCA, HNSC, LIHC, LUAD, TGCT, and UCEC but higher in ESCA, KIRC, KIRP, and PAAD (Figure 6A). This suggested that DNA methylation levels affect gene expression. As shown in Figure 6B, NCAPD2 expression was positively correlated with the expression of RNA methylation-regulated genes in most cancers.

### NCAPD2 is associated with ICB response

**Figure 7:**
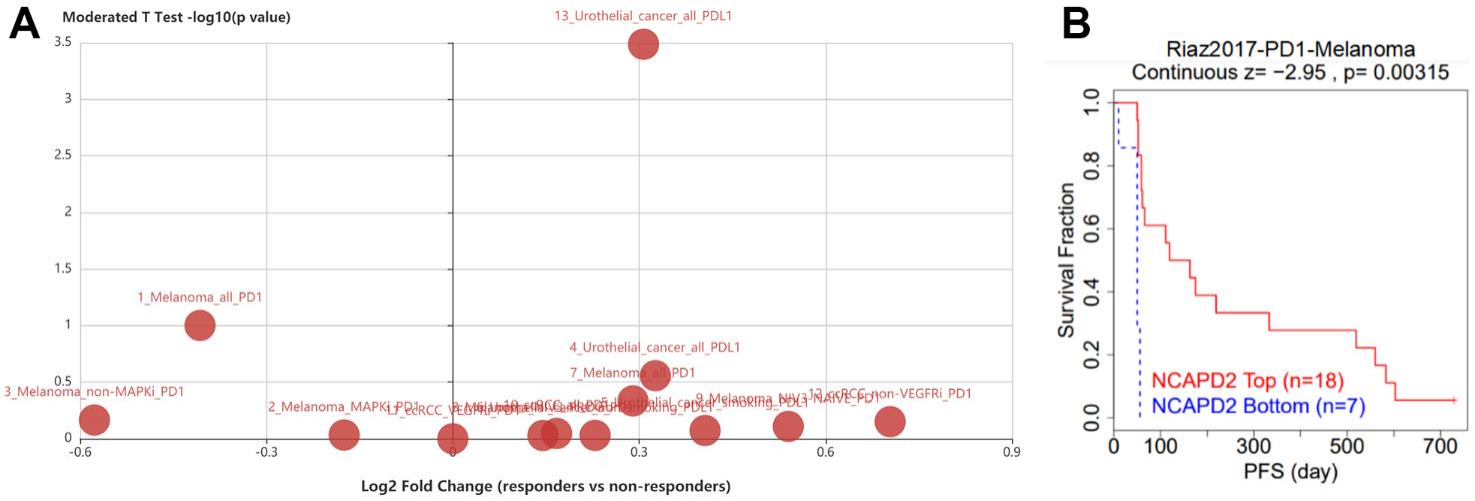
(**A**) NCAPD2 expression was higher in responders than in nonresponders in urothelial carcinoma patients treated with PD-L1 inhibitor atezolizumab. (**B**) The Kaplan-Meier survival of NCAPD2 in low- and high-expression treated with PD-1 in melanoma.

The TISIDB database showed that NCAPD2 expression was higher in responders than in nonresponders in urothelial carcinoma treated with the PD-L1 inhibitor atezolizumab (Figure 7A). We utilized the TIDE database to analyze the AUC of NCAPD2. We compared it with existing biomarkers, such as TMB and MSI, to predict immunotherapy response. NCAPD2 showed good predictive ability (AUC > 0.7) in HNSC and melanoma, MSI showed good predictive ability (AUC > 0.7) in melanoma and gastric cancer, and TMB had good predictive ability (AUC > 0.7) in urothelial cancer. Notably, NCAPD2 was the only biomarker that achieved good performance in terms of ICB response in HNSC. TMB was used as a biomarker in bladder cancer (AUC = 0.73) in predicting ICB response, whereas MSI was used as a similar biomarker in gastric cancer (AUC = 0.75) (Table 1). Moreover, in the melanoma cohort, highly expressed NCAPD2 was associated with a better prognosis after treatment with PD-1 inhibitors (Figure 7B). Therefore, NCAPD2 can be used as a biomarker to predict immunotherapy response for a certain tumor.

**Table 1:** The biomarker evaluation and AUC of NCAPD2 in response to immunotherapy in cancer using TIDE database.

**Study**	**Cancer type**	**Treatment**	**AUC (NCAPD2)**	**MSI score**	**TMB**
Zhao, 2019	Glioblastoma	PD-1-Pre	0.41	0.41	n
		PD-1-Post	0.22	0.67	n
Van Allen, 2015	Melanoma	CTLA4	0.47	0.74	0.67
Uppaluri, 2020	HNSC	PD1-Pre	0.61	0.63	n
		PD-1-Post	0.79	0.45	n
Ruppin, 2021	NSCLC	PD-1	0.52	0.46	n
Riaz, 2017	Melanoma	PD-1-Prog	0.41	0.70	0.47
		PD-1-Naive	0.72	0.40	0.62
Nathanson, 2017	Melanoma	CTLA4-Pre	0.15	0.95	n
		CTLA4-Post	0.545	0.52	n
Miao 2018	RCC	ICB	0.55	0.25	0.65
McDermott 2018	RCC	PDL1	0.45	0.55	0.54
Mariathasan 2018	Bladder	PDL1-mUC	0.63	0.56	0.73
Liu, 2019	Melanoma	PD1-prog	0.58	0.45	n
		PD1-Naive	0.63	0.50	n
Kim, 2018	Gastric	PD1	0.52	0.75	n
Hugo, 2016	Melanoma	PD1	0.29	0.69	n
Gide, 2019	Melanoma	PD1	0.61	0.43	0.63
Gide, 2019	Melanoma	PD1+CTLA4	0.44	0.70	n
Braun, 2020	RCC	PD1	0.51	0.53	0.56

### Drug sensitivity analysis of NCAPD2

**Figure 8:**
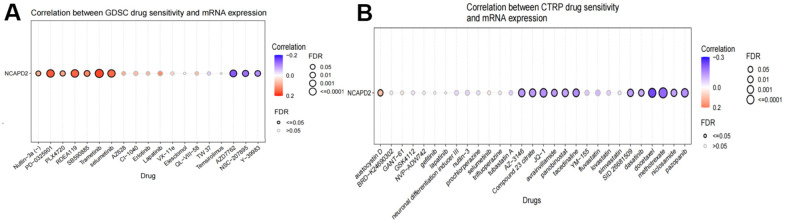
Correlation between drug sensitivity and NCAPD2 expression in the (**A**) GDSC and (**B**) CTRP database.

Multiple drugs that were sensitive to NCAPD2 were predicted using the GDSC and CTRP datasets from the GSCA database. In the GDSC dataset, there was a negative correlation between NCAPD2 and 50% inhibitory concentration (IC50) values of AZD7762, NSC-207895, and Y-39983. In the CTRP dataset, there was a negative correlation between NCAPD2 and the IC50 values of 12 types of drugs: methotrexate, docetaxel, pazopanib, SID26681509, dasatinib, niclosamide, JQ-1, compound 23 citrate, AZ-3146, avrainvillamide, panobinostat, and tacedinaline. These results indicate that NCAPD2 is significantly related to different drug sensitivities in different tumor cell lines and may be a promising cancer treatment target (Figure 8).

### NCAPD2 co-expression genes’ enrichment analysis

**Figure 9:**
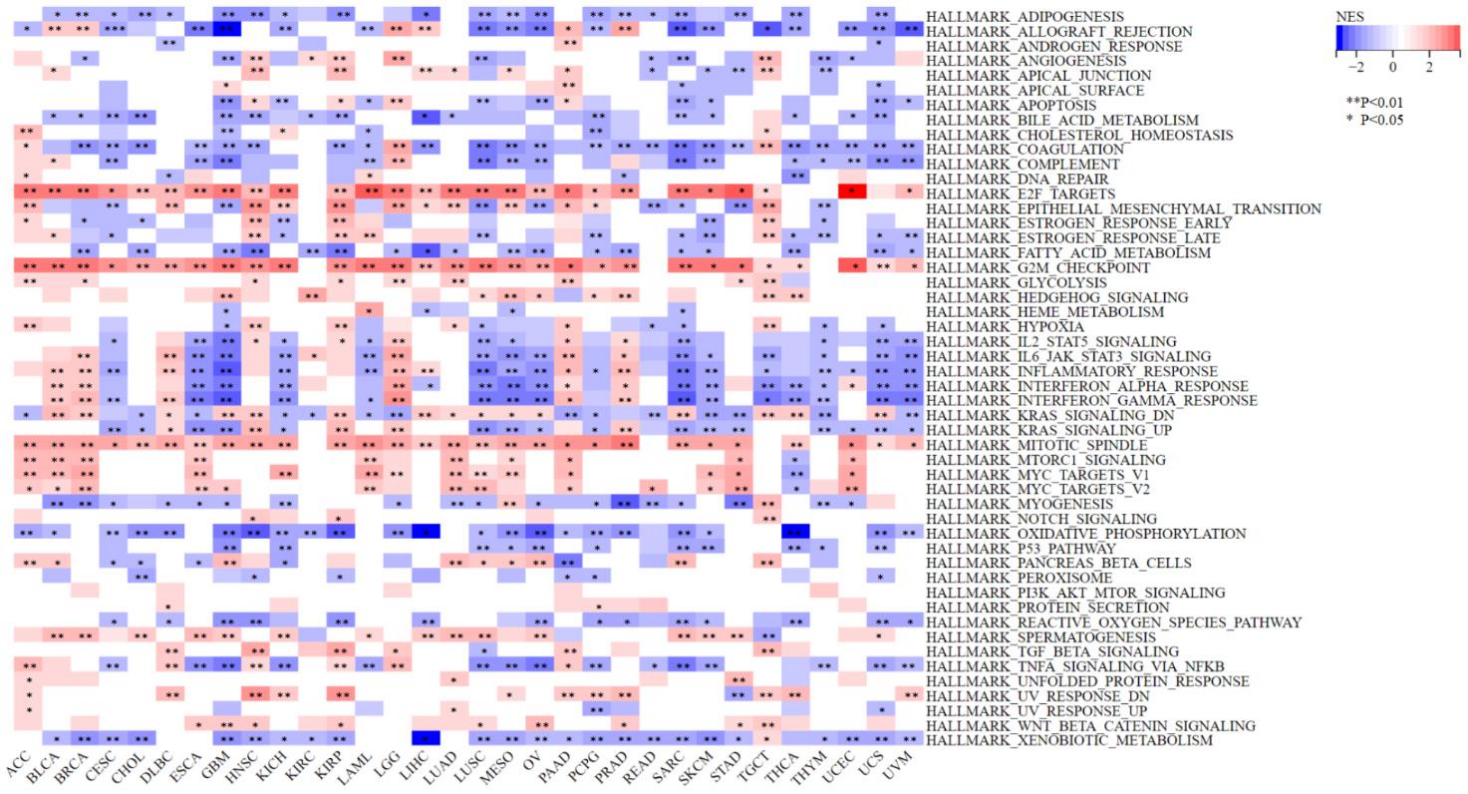
**Gene set enrichment analysis of NCAPD2 in high- and low-expression groups for 33 cancers.** (***P < 0.001,**P < 0.01,*P < 0.05).

GSEA results showed that NCAPD2 was involved mainly in the following pathways in the vast majority of tumors: G2M-checkpoint, mitotic spindle, KRAS-signaling, E2F-target, coagulation, and xenobiotic metabolism. These findings indicate that NCAPD2 may be associated with tumorigenesis (Figure 9).

### Overexpression NCAPD2 was an independent biomarker in LIHC and its correlation with clinical features

**Figure 10:**
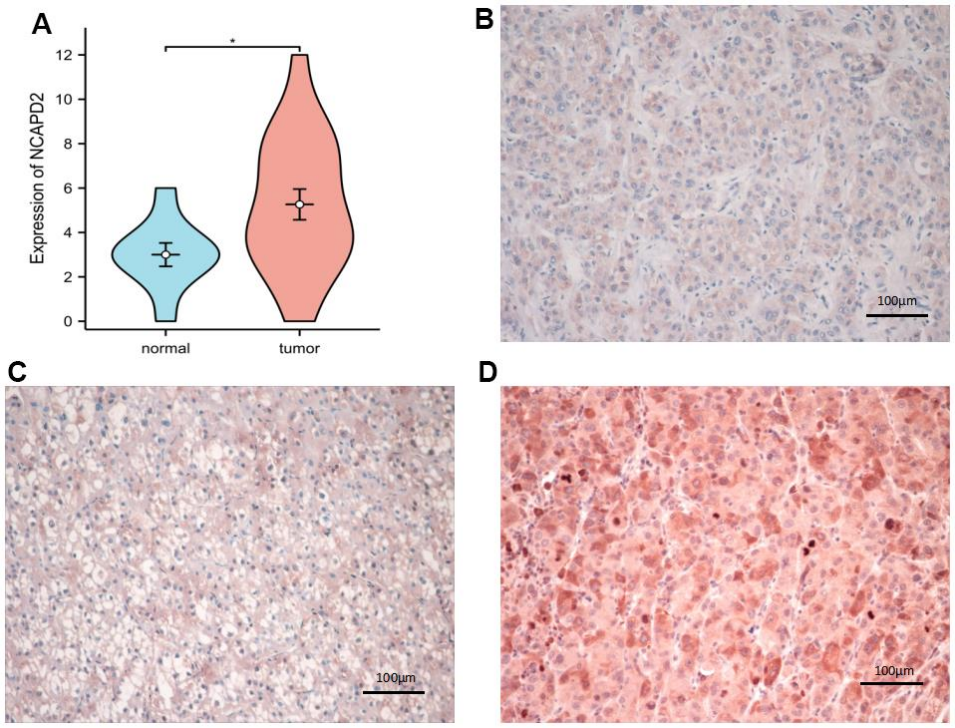
**The expression of NCAPD2 protein in normal tissues and LIHC by immunohistochemistry (EnVision; original magnification, ×200).** (**A**) NCAPD2 had higher expression in LIHC than normal tissues. (**B**) Weak positive expression of NCAPD2 in LIHC. (**C**) Moderate positive expression of NCAPD2 in LIHC. (**D**) Strong positive expression of NCAPD2 in LIHC. (*P<0.05).

**Figure 11:**
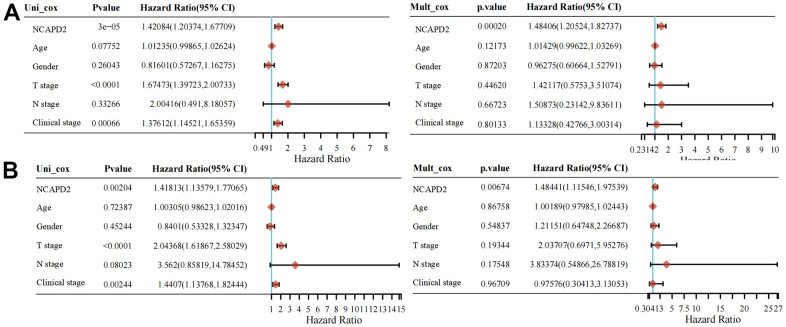
Univariate and multivariate Cox regression of NCAPD2 for OS (**A**) and DSS (**B**).

**Figure 12:**
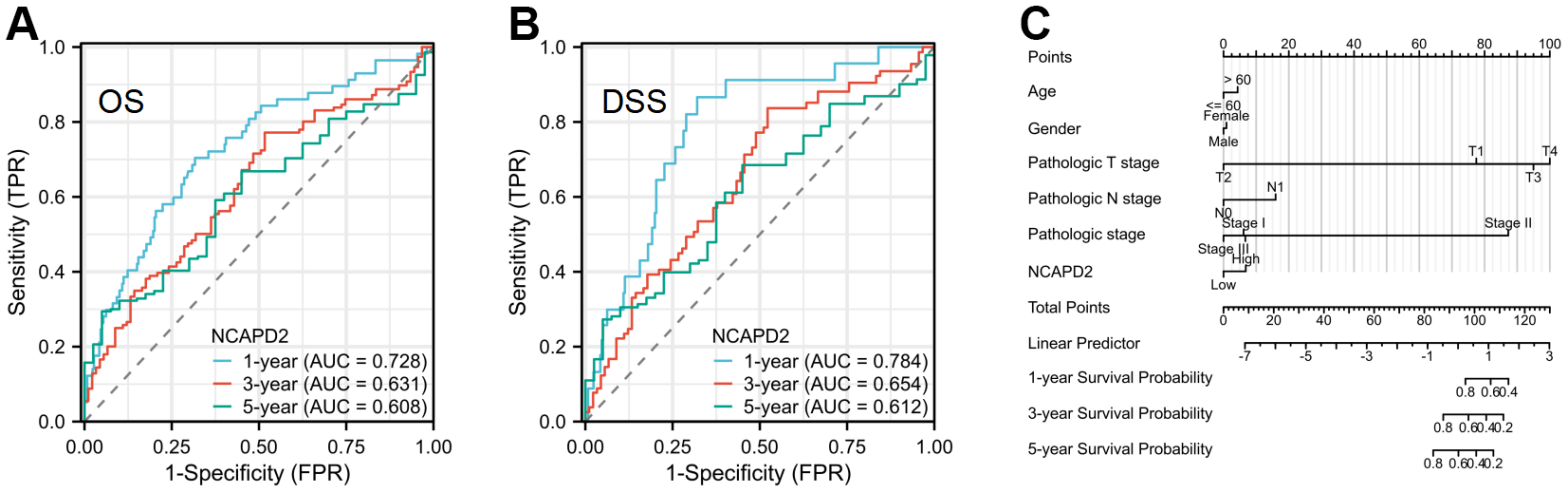
**The time ROC curve of 1-, 3-, and 5-year OS and DSS in LIHC patients, and prognostic nomogram.** (**A**) The time ROC curve of 1-, 3-, and 5-year OS in LIHC. (**B**) The time ROC curve of 1-, 3-, and 5-year DSS in LIHC. (**C**) The prognostic nomogram of NCAPD2 in LIHC.

To further confirm the difference in NCAPD2 protein expression in tumors and normal tissues, we chose twenty cases of LIHC and their corresponding normal samples for IHC. IHC scores showed that NCAPD2 had higher expression in LIHC than in normal tissues (Figure 10A–10D). Univariate and multivariate Cox regression analysis demonstrated NCAPD2 was an independent biomarker in predicting prognosis for OS and DSS (Figure 11A, 11B). We studied the relationship between NCAPD2 and various clinical subgroups in LIHC. The results showed that NCAPD2 expression was associated with T stage (P=0.008), pathologic stage (P=0.006), and histologic grade (P<0.001). Moreover, we studied the relationship of NCAPD2 expression with the prognosis of OS in various clinical subgroups of LIHC (Table 2). The time ROC curve demonstrated that 1-, 3-, and 5-year of OS were 0.728, 0.631, and 0.608(Figure 12A). The 1-, 3-, and 5-year of DSS were 0.784, 0.654, and 0.612 (Figure 12B). Moreover, we drew the nomogram for predicting 1-, 3-, and 5-year OS and DSS (Figure 12C). These findings suggested NCAPD2 had good predictive value.

**Table 2:** The association between NCAPD2 and clinical features in LIHC patients.

**Type**	**NCAPD2 low expression**	**NCAPD2 high expression**	**P value**
n	187	187	
Gender			0.060
Female	52	69	
Male	135	118	
Pathologic T stage			**0.008**
T1&T2	149	129	
T3&T4	35	58	
Pathologic N stage			0.727
N0	118	136	
N1	1	3	
Pathologic M stage			0.573
M0	129	139	
M1	3	1	
Pathologic stage			**0.006**
Stage I&Stage II	139	121	
Stage III&Stage IV	33	57	
Histologic grade			**< 0.001**
G1&G2	138	95	
G3&G4	47	89	

## DISCUSSION

The condensin complex is a multi-protein complex that not only plays a central role in chromatin condensation nuclear segregation during mitosis and meiosis but also participates in the maintenance of genome stability and cell differentiation, including condensing complex I and condensing complex II. NCAPD2 belongs to the condensing complex I and is located on chromosome 12p13.31, which is universally expressed in whole-body tissues and plays a role in chromosome condensation. NCAPD2 plays a pivotal role in the occurrence and development of tumors and non-tumorous diseases. Some reports have found that NCAPD2 is linked to several neurodegenerative diseases, such as Alzheimer’s disease and Parkinson’s disease, indicating the effect of NCAPD2 in the development of the central nervous system [6, 23]. Overexpression of NCAPD2 promotes the release of proinflammatory substances through the regulation of the IKK/NF-κB signaling pathway, which is also involved in the pathogenesis of colitis [24]. Furthermore, NCAPD2 is overexpressed in breast cancer and has been implicated in breast cancer prognosis. NCAPD2 knockdown inhibits breast cancer progression by inhibiting proliferation, migration, and apoptosis *in vitro* and *in vivo*. Conversely, its expression promoted breast cancer progression by interacting with E2F transcription factor 1 (E2F1), resulting in CDK1 activation in MDA-MB-231 cells [25]. NCAPD2 is also upregulated in ovarian cancer, wherein amplification and mutations have been observed [26]. NCAPD2 is similarly upregulated in colorectal cancer and NCAPD2 inhibits autophagy and promotes CRC cell proliferation and migration through the Ca2+/CAMKK/AMPK/mTORC1 pathway and PARP-1/SIRT1 axis [27]. Therefore, we conducted a pan-cancer analysis of NCAPD2 gene expression, survival status, immune activity, DNA methylation, RNA methylation, and related cellular pathways based on multiple public databases.

In our study, the overexpression of NCAPD2 in the vast majority of cancers was confirmed by TCGA and GTEx databases. NCAPD2 protein overexpression in BRCA, COAD, GBM, LIHC, HNSC, LUAD, PAAD, KIRC, and UCEC was confirmed in the CPTAC database. Moreover, using the HPA database, it was found that NCAPD2 expression was higher in colon, breast, and lung cancers than in normal tissues. We analyzed the correlation between NCAPD2 levels and clinical features. Interestingly, NCAPD2 expression was higher in the non-Asian group than in the Asian group for BLCA. Conversely, NCAPD2 expression was lower in the non-Asian group for BRCA, ESCA, KIRC, and LIHC. The expression of NCAPD2 was correlated with T, N, and pathological stages in some tumors. These findings illustrated the importance of NCAPD2 in tumor progression. Furthermore, we evaluated the effect of NCAPD2 expression on the prognosis of cancer patients. NCAPD2 overexpression was associated with poor prognosis with OS, and DSS in ACC, KIRP, LGG, LIHC, LUAD, MESO, PAAD, SARC, SKCM, and UCEC. The ROC of NCAPD2 was above 0.8 in 20 types of cancer, indicating a better diagnostic ability to distinguish between tumor tissue and normal tissue.

TME is defined as the microenvironment associated with tumor cell growth, proliferation, and metastasis. Therefore, we investigated the crucial role of NCAPD2 in cancer initiation and progression. NCAPD2 expression had a significantly positive association with the six immune cells for KIRC, LGG, LAML, PCPG, PRAD, and THCA, and negative association with the six immune cells in TGCT. These results demonstrate that NCAPD2 may play a role in the TME. Furthermore, we used the ESTIMATE algorithm to estimate the proportion of immune cells and stromal cells in tumors. In our study, NCAPD2 had a negative relationship with stromal, immune, and estimated scores for 10 tumors, a positive correlation for two tumors, and a positive or negative association with stromal or immune scores in other tumors. Subsequently, we investigated the relationship between NCAPD2 and immune modulators and found that most tumors were positively linked to immune regulatory genes. With the emergence of immune checkpoints, ICIs have emerged, bringing hope to the immunotherapy of tumors. We researched the correlation of NCAPD2 with immune checkpoints (ICs) for various tumors and found that except for ACC, CESC, ESCA, LAML, MESO, and UVM, the expression of NCAPD2 in the remaining tumors was correlated with one or more immune checkpoints. For example, NCAPD2 expression was positively associated with eight ICs in LIHC and KIRC, suggesting that high NCAPD2 expression in LIHC and KIRC may benefit from ICIs treatment. These findings indicate that NCAPD2 may serve as a latent marker that influences the effects of immune therapy.

Cancer immunotherapy technology has made significant progress in clinical practice and has been widely used in the treatment of a variety of malignant tumors, but only some patients respond and benefit from it. Therefore, finding potential markers has become the key to immunotherapy [28]. TMB and MSI are two effective prognostic markers and predictors of immunotherapeutic response in the majority of tumors [10, 11, 29]. Patients with high TMB and high MSI often have better objective response rates to immunotherapy and longer clinical survival [30–32]. In this study, NCAPD2 was found to be positively linked with TMB in nine types of cancer and negatively correlated with TMB in three types of cancer. It can be speculated that the highly expressed NCAPD2 in nine cancers has a high TMB and better immunotherapy response [33]. NCAPD2 was positively associated with MSI in eight tumors and negatively correlated in two tumors. This indicated that NCAPD2, highly expressed in eight tumors, had high MSI, which was conducive to the efficacy of immunotherapy. Thus, NCAPD2 expression affected the TMB and MSI of tumors, thereby influencing the outcome of tumor immunotherapy. Through TISIDB, we found that NCAPD2 expression in responders was higher than that in nonresponders in urothelial carcinoma treated with the PD-L1 inhibitor, atezolizumab. TIDE analysis showed that NCAPD2 is a better biomarker to achieve good performance in ICB response in HNSC than MSI and TMB. In the melanoma cohort, NCAPD2 with high expression was associated with a better prognosis after anti-PD-1 therapy. Therefore, NCAPD2 may be a promising biomarker for immunological therapy in certain cancers. We also studied the correlation between NCAPD2 and drug sensitivity using the GSCA database. NCAPD2 had a negative correlation with 50% inhibitory concentration (IC50) values of the three drugs in the GDSC dataset and 12 types of drugs in the CTRP dataset. The *in vivo* effects of antitumor drugs need to be further verified. Therefore, targeting NCAPD2 may be a promising strategy for cancer treatment.

DNA methylation and RNA methylation have become the most common and widespread epigenetic pathways, and their methylation levels have been found to affect gene expression in malignant tumors and serve as a predictive biomarker for diagnosis and personalized therapy [34, 35]. In this study, a relevance analysis was conducted for NCAPD2 expression and promoter methylation levels using the UALCAN tool. The results showed that compared with the normal tissue, NCAPD2 promoter methylation levels decreased significantly in BLCA, BRCA, HNSCC, LIHC, LUAD, TGCT, and UCEC. This may explain the overexpression of NCAPD2 in various cancers. However, NCAPD2 methylation levels were higher in ESCA, KIRC, KIRP, and PAAD tissues than in normal tissues. Our results showed that NCAPD2 expression was positively correlated with the expression of RNA methylation-regulated genes in the majority of cancers. This is also the first study to reveal a relationship between NCAPD2 and methylation, which requires further experimental verification.

To further elucidate the role of NCAPD2 in the biological processes in various tumors, we classified NCAPD2 expression into high- and low-expression groups for GSEA analysis. NCAPD2 chiefly participates in the signaling pathway of the G2M-checkpoint, mitotic spindle, KRAS-signaling, E2F-target, coagulation, and xenobiotic metabolism. This indicates that NCAPD2 caused tumorigenesis through the above pathways, which requires further experiments.

We selected liver cancer for the IHC study and we found that NCAPD2 was up-regulated in LIHC and NCAPD2 expression was related to T stage, pathologic stage, and histologic grade. NCAPD2 was an independent marker in the prognosis of OS and DSS by univariate and multivariate analysis. In addition, the time ROC curve showed that 1-, 3-, and 5-year OS and DSS were above 0.6, which was a good predictive marker.

However, our study had some limitations. We studied NCAPD2 protein expression in LIHC only by IHC. The analysis and results of this study are obtained from publicly available datasets, and some of the findings require further efforts to validate them experimentally. In addition, there are few studies on NCAPD2, and more explanation from experimental studies is needed.

## CONCLUSIONS

In our study, NCAPD2 expression was systematically analyzed in 33 tumors and correlated with the clinical stage of some tumors, which can be regarded as a valuable biomarker for prognosis in many tumors. In addition, NCAPD2 was significantly linked with TME, immune checkpoints, TMB, MSI, DNA methylation, and RNA methylation in a variety of tumors, which contributed to the understanding of the latent role of NCAPD2 in human cancers from multiple viewpoints. The mechanism of action of NCAPD2 in tumors requires further experimental studies.

## Supplementary Material

Supplementary Figure 1

Supplementary Table 1
