# Supplementary material for: Non-SMC condensin I complex subunit D2 (NCAPD2) reveals its prognostic and immunologic features in human cancers
Source: Aging (Albany NY). 2023 Jul 26;15(14):7237–57. doi: 10.18632/aging.204904 (PMC10415567; doi:10.18632/aging.204904)
Supplement: Supplementary Figure 1 [file aging-15-204904-s001.pdf]

SUPPLEMENTARY FIGURE

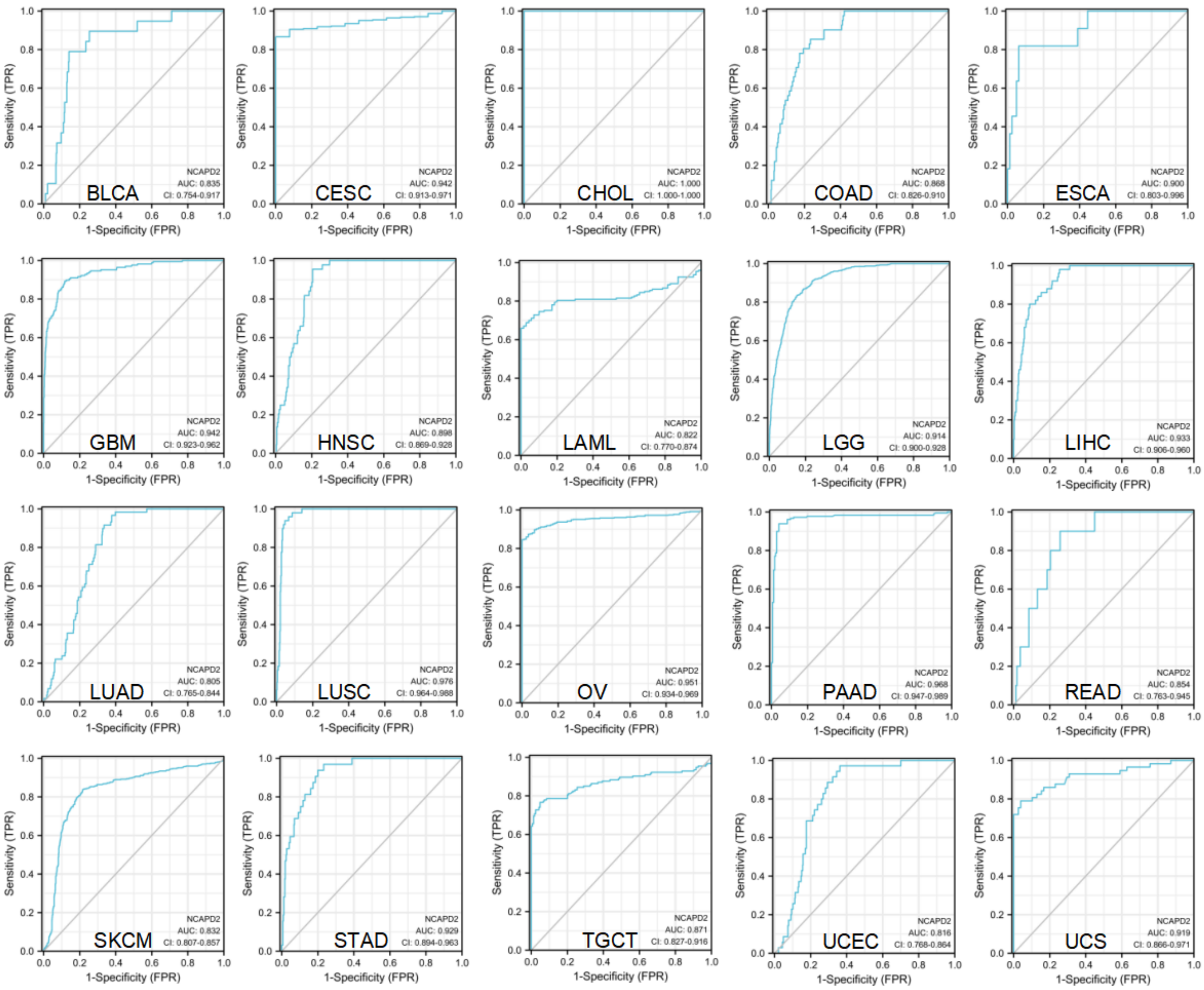

Supplementary Figure 1. The ROC curve of NCAPD2 in 20 types of tumors, distinguishing cancers from normal tissues.
